# Anharmonic Vibrational Frequencies of Water Borane and Associated Molecules

**DOI:** 10.3390/molecules26237348

**Published:** 2021-12-03

**Authors:** Brent R. Westbrook, Ryan C. Fortenberry

**Affiliations:** Department of Chemistry & Biochemistry, University of Mississippi, University, MS 38677, USA; bwestbr2@go.olemiss.edu

**Keywords:** vibrational spectroscopy, anharmonic frequencies, rotational spectroscopy, quantum chemistry, alternative fuels, coupled cluster theory, hydrogen production

## Abstract

Water borane (BH${}_{3}$OH${}_{2}$) and borinic acid (BH${}_{2}$OH) have been proposed as intermediates along the pathway of hydrogen generation from simple reactants: water and borane. However, the vibrational spectra for neither water borane nor borinic acid has been investigaged experimentally due to the difficulty of isolating them in the gas phase, making accurate quantum chemical predictions for such properties the most viable means of their determination. This work presents theoretical predictions of the full rotational and fundamental vibrational spectra of these two potentially application-rich molecules using quartic force fields at the CCSD(T)-F12b/cc-pCVTZ-F12 level with additional corrections included for the effects of scalar relativity. This computational scheme is further benchmarked against the available gas-phase experimental data for the related borane and HBO molecules. The differences are found to be within 3 cm${}^{-1}$ for the fundamental vibrational frequencies and as close as 15 MHz in the $B_{0}$ and $C_{0}$ principal rotational constants. Both BH${}_{2}$OH and BH${}_{3}$OH${}_{2}$ have multiple vibrational modes with intensities greater than 100 km mol${}^{-1}$, namely $\nu_{2}$ and $\nu_{4}$ in BH${}_{2}$OH, and $\nu_{1}$, $\nu_{3}$, $\nu_{4}$, $\nu_{9}$, and $\nu_{13}$ in BH${}_{3}$OH${}_{2}$. Finally, BH${}_{3}$OH${}_{2}$ has a large dipole moment of 4.24 D, which should enable it to be observable by rotational spectroscopy, as well.

## 1. Introduction

Borane-containing molecules like ammonia borane are promising hydrogen storage media for use in fuel cells due to their high hydrogen density [1,2,3,4]. However, all of this storage capacity is of little use without a clean way to liberate the hydrogen into hydrogen gas. To this end, recent work has revived an interest in borane as a feedstock for generating hydrogen gas from water that dates back at least to the 1950s [5,6]. Combining borane [7] or diborane [5,7] with water can produce substantial amounts of hydrogen gas, which is becoming increasingly important as a source of alternative fuels [6,8,9,10,11]. Current methods of producing hydrogen gas, however, still rely primarily upon fossil fuels, limiting the clean nature of the resulting hydrogen [7]. As shown previously [6,7], an important step along the hydrogen production pathway when using borane feedstocks is the formation of BH${}_{3}$OH${}_{2}$ or water borane. This can then decompose with a submerged barrier into one equivalent of hydrogen gas and borinic acid, BH${}_{2}$OH. Such a pathway suggests that borane and its hydrated or ammonia-complexed variants may have a substantial role to play not only in the storage of large amounts of hydrogen but also in the generation of hydrogen from water.

Limiting the optimism surrounding such promise is the difficulty of isolating and then conclusively identifying individual borane complexes in the gas phase. Such identification is of the utmost necessity given the fact that borane tends to form dative bonds that are exquisitely sensitive to the surrounding environment [12,13]. In ammonia borane, in particular, this spectroscopic sensitivity has led theoretical work on the ammonia borane dimer to show shifts of up to 40 cm${}^{-1}$ in the B-N stretching frequency from the isolated molecule [12]. Similarly, IR and Raman experiments exhibit even larger shifts of up to 150 cm${}^{-1}$ in the solid phase [14,15]. While previous work has demonstrated that boron forms stronger bonds to oxygen than it does to nitrogen [16], these bonds will likely still be dative and preserve the same sensitivity observed in the B-N bond of ammonia borane. In both cases, this behavior means these molecules, and the boron-heavy atom stretching frequency in particular, can also serve as indicators of their environmental conditions. Better knowledge of these conditions may help to inform designers of water splitting catalysts of the H${}_{2}$-producing mechanism. For these indicators to be useful, however, there must be highly accurate, benchmark vibrational data for the isolated molecules.

Unfortunately, the same sensitivity that makes such data appealing also increases the difficulty of obtaining it experimentally. As a result, theoretical investigations are the best chance for obtaining accurate vibrational data for these sensitive frequencies. Previous work [13] on ammonia borane demonstrates agreement in the computed values to within 5 cm${}^{-1}$ of the seven available gas-phase vibrational frequencies [4] and offers a new theoretical prediction of the B-N stretching frequency that continues to elude experimental detection.

The previous work on ammonia borane [13] utilizes a quartic force field (QFF) methodology combined with coupled cluster theory at the singles, doubles, and perturbative triples level [17] within the F12 explicitly correlated construction (CCSD(T)-F12b) [18,19] and a triple-ζ basis set. Such a method and basis set combination is commonly abbreviated as F12-TZ. QFFs are fourth-order Taylor series expansions of the internuclear potential energy portion of the Watson Hamiltonian [20]. When coupled with the F12-TZ level of theory, QFFs frequently offer agreement with gas-phase vibrational frequencies of within 5 to 7 cm${}^{-1}$ [21,22,23,24,25]. Other techniques for computing accurate anharmonic spectral data exist [26], but as a result of the good performance of the F12-TZ QFF on ammonia borane, this same methodology is used herein to investigate water borane (BH${}_{3}$OH${}_{2}$), borinic acid (BH${}_{2}$OH), HBO, and borane (BH${}_{3}$).

One shortcoming of the F12-TZ methodology is its inability to produce very accurate rotational constants [22,23,24]. When accurate rotational data is needed, much more expensive composite QFFs have often been employed that achieve agreement of about 20 MHz in the vibrationally-averaged ground state rotational constants [27]. Chief among these composite methods is CcCR, which is composed of a complete basis set extrapolation (“C”), corrections for core correlation (“cC”), and corrections for scalar relativity (“R”) [20]. However, recent work [28] has explored the use of a hybrid between F12-TZ and CcCR, fittingly referred to as F12-TZ-cCR. This method utilizes CCSD(T)-F12b with the cc-pCVTZ-F12 basis set, explicit treatment of core electrons, and the same correction for scalar relativity as CcCR. It offers more accurate rotational constants with agreement on the order of 7.5 MHz with experimental data while still capturing an order-of-magnitude decrease in the computational cost relative to CcCR [28]. As such, this methodology is also employed herein to offer better predictions of the rotational spectra of these molecules.

While water borane and borinic acid do not have existing gas-phase infrared data, the related borane and HBO molecules do. Kawaguchi et al. have reported high-resolution vibrational frequencies for borane [29], and Kawashima et al. have determined both rotational and vibrational experimental data for HBO [30]. In both cases, these data will help to benchmark the accuracy of the theoretical results presented herein on the structurally similar water borane and borinic acid molecules. Borinic acid also has some available rotational constants [31] that will further help to contextualize the rotational data reported here, as well.

## 2. Computational Details

The F12-TZ and F12-TZ-cCR computations performed in the present work, including geometry optimizations, harmonic frequencies, dipoles, and single-point energies, all utilize the Molpro 2020.1 software package [32]. All of the F12-TZ computations solely use the cc-pVTZ-F12 basis set [21,33,34], while the F12-TZ-cCR geometry optimizations require the cc-pCVTZ-F12 basis set [34]. The F12-TZ-cCR single-point energy computations additionally utilize canonical CCSD(T) with a cc-pVTZ-DK basis set to account for the effects of scalar relativity [35,36]. Double-harmonic and anharmonic infrared intensities are computed within the Gaussian16 suite of programs [37] using the MP2/aug-cc-pVTZ level of theory [38,39]. The harmonic values at this level of theory have been previously shown to yield semi-quantitative accuracy in the infrared intensities, and the differences from the anharmonic values are typically negligible [40,41,42].

**Figure 1:**
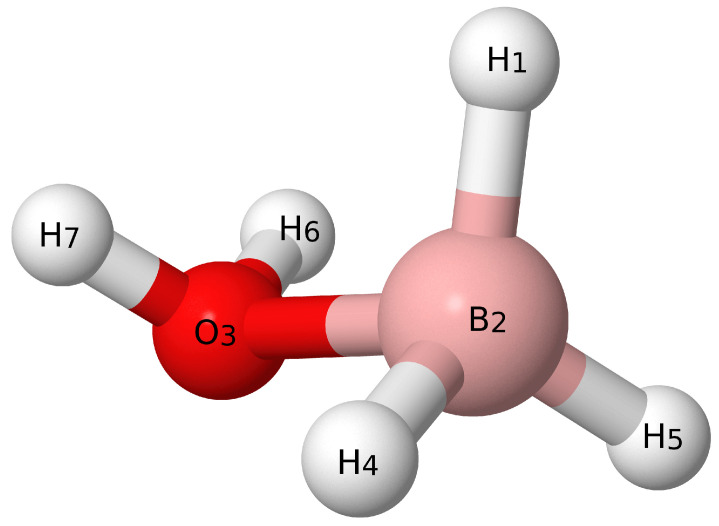
Visual depiction of BH${}_{3}$OH${}_{2}$.

For both the F12-TZ and F12-TZ-cCR QFFs, following the geometry optimization, displacements of 0.005 Å or radians are taken from the optimized geometry to map out the QFF. The symmetry internal coordinates (SICs) along which these displacements are taken are shown below for BH${}_{3}$OH${}_{2}$ with atom labels corresponding to Figure 1.

(1) $$\begin{array}{ccc} S_{1}\left(a^{\prime }\right) & = & r(H_{1}-B_{2}) \end{array}$$

(2) $$\begin{array}{ccc} S_{2}\left(a^{\prime }\right) & = & r(B_{2}-O_{3}) \end{array}$$

(3) $$\begin{array}{ccc} S_{3}\left(a^{\prime }\right) & = & \frac{1}{\sqrt{2}}\left[r\left(B_{2}-H_{4}\right)+r\left(B_{2}-H_{5}\right)\right] \end{array}$$

(4) $$\begin{array}{ccc} S_{4}\left(a^{\prime }\right) & = & \frac{1}{\sqrt{2}}\left[r\left(O_{3}-H_{6}\right)+r\left(O_{3}-H_{7}\right)\right] \end{array}$$

(5) $$\begin{array}{ccc} S_{5}\left(a^{\prime }\right) & = & ∠(H_{1}-B_{2}-O_{3}) \end{array}$$

(6) $$\begin{array}{ccc} S_{6}\left(a^{\prime }\right) & = & \frac{1}{\sqrt{2}}\left[∠\left(H_{5}-B_{2}-O_{3}\right)+∠\left(H_{4}-B_{2}-O_{3}\right)\right] \end{array}$$

(7) $$\begin{array}{ccc} S_{7}\left(a^{\prime }\right) & = & \frac{1}{\sqrt{2}}\left[∠\left(H_{6}-O_{3}-B_{2}\right)+∠\left(H_{7}-O_{3}-B_{2}\right)\right] \end{array}$$

(8) $$\begin{array}{ccc} S_{8}\left(a^{\prime }\right) & = & \frac{1}{\sqrt{2}}\left[\tau \left(H_{1}-B_{2}-O_{3}-H_{6}\right)-\tau \left(H_{1}-B_{2}-O_{3}-H_{7}\right)\right] \end{array}$$

(9) $$\begin{array}{ccc} S_{9}\left(a^{\prime }\right) & = & \frac{1}{\sqrt{2}}\left[\tau \left(H_{5}-B_{2}-O_{3}-H_{6}\right)-\tau \left(H_{4}-B_{2}-O_{3}-H_{7}\right)\right] \end{array}$$

(10) $$\begin{array}{ccc} S_{10}\left(a^{\prime \prime }\right) & = & \frac{1}{\sqrt{2}}\left[r\left(B_{2}-H_{4}\right)-r\left(B_{2}-H_{5}\right)\right] \end{array}$$

(11) $$\begin{array}{ccc} S_{11}\left(a^{\prime \prime }\right) & = & \frac{1}{\sqrt{2}}\left[r\left(O_{3}-H_{6}\right)-r\left(O_{3}-H_{7}\right)\right] \end{array}$$

(12) $$\begin{array}{ccc} S_{12}\left(a^{\prime \prime }\right) & = & \frac{1}{\sqrt{2}}\left[∠\left(H_{5}-B_{2}-O_{3}\right)-∠\left(H_{4}-B_{2}-O_{3}\right)\right] \end{array}$$

(13) $$\begin{array}{ccc} S_{13}\left(a^{\prime \prime }\right) & = & \frac{1}{\sqrt{2}}\left[∠\left(H_{6}-O_{3}-B_{2}\right)-∠\left(H_{7}-O_{3}-B_{2}\right)\right] \end{array}$$

(14) $$\begin{array}{ccc} S_{14}\left(a^{\prime \prime }\right) & = & \frac{1}{\sqrt{2}}\left[\tau \left(H_{1}-B_{2}-O_{3}-H_{6}\right)+\tau \left(H_{1}-B_{2}-O_{3}-H_{7}\right)\right] \end{array}$$

(15) $$\begin{array}{ccc} S_{15}\left(a^{\prime \prime }\right) & = & \frac{1}{\sqrt{2}}\left[\tau \left(H_{5}-B_{2}-O_{3}-H_{6}\right)+\tau \left(H_{4}-B_{2}-O_{3}-H_{7}\right)\right] \end{array}$$

**Figure 2:**
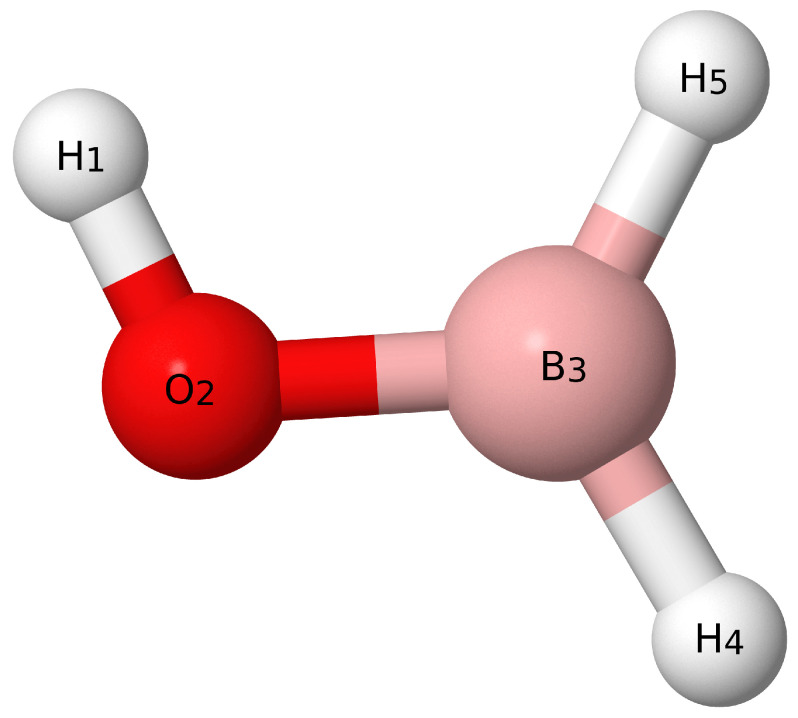
Visual depiction of borinic acid.

Similarly, the SICs for borinic acid with atom labels given by Figure 2 are

(16) $$\begin{array}{ccc} S_{1}\left(a^{\prime }\right) & = & r(H_{1}-O_{2}) \end{array}$$

(17) $$\begin{array}{ccc} S_{2}\left(a^{\prime }\right) & = & r(O_{2}-B_{3}) \end{array}$$

(18) $$\begin{array}{ccc} S_{3}\left(a^{\prime }\right) & = & r(B_{3}-H_{4}) \end{array}$$

(19) $$\begin{array}{ccc} S_{4}\left(a^{\prime }\right) & = & r(B_{3}-H_{5}) \end{array}$$

(20) $$\begin{array}{ccc} S_{5}\left(a^{\prime }\right) & = & ∠(H_{1}-O_{2}-B_{3}) \end{array}$$

(21) $$\begin{array}{ccc} S_{6}\left(a^{\prime }\right) & = & ∠(O_{2}-B_{3}-H_{4}) \end{array}$$

(22) $$\begin{array}{ccc} S_{7}\left(a^{\prime }\right) & = & ∠(O_{2}-B_{3}-H_{5}) \end{array}$$

(23) $$\begin{array}{ccc} S_{8}\left(a^{\prime \prime }\right) & = & \tau (H_{1}-O_{2}-B_{3}-H_{4}) \end{array}$$

(24) $$\begin{array}{ccc} S_{9}\left(a^{\prime \prime }\right) & = & \tau (H_{1}-O_{2}-B_{3}-H_{5}), \end{array}$$

**Figure 3:**
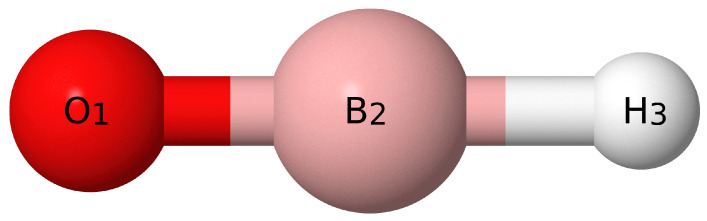
Visual depiction of HBO.

Those for HBO with atom labels from Figure 3 are

(25) $$\begin{array}{ccc} S_{1}\left(\sigma \right) & = & r(O_{1}-B_{2}) \end{array}$$

(26) $$\begin{array}{ccc} S_{2}\left(\sigma \right) & = & r(B_{2}-H_{3}) \end{array}$$

(27) $$\begin{array}{ccc} S_{3}/S_{4}\left(\pi \right) & = & ∠(O_{1}-B_{2}-H_{3}), \end{array}$$

**Figure 4:**
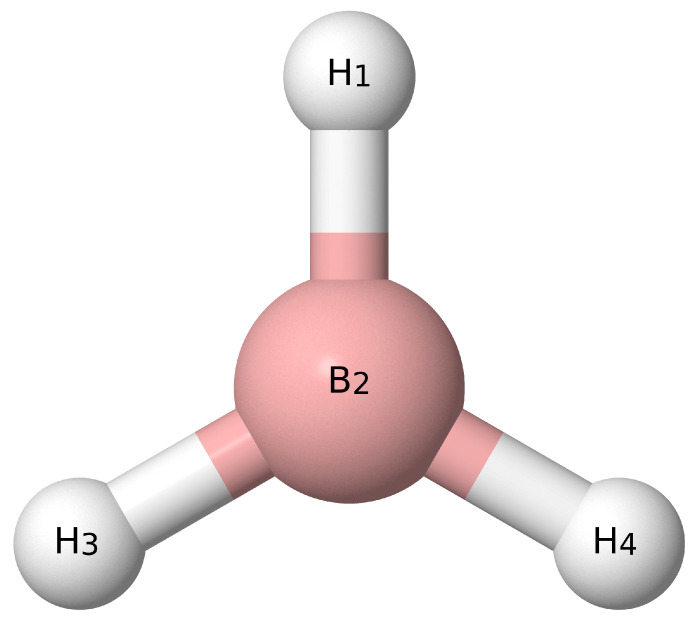
Visual depiction of borane.

And those for borane with atom labels from Figure 4 are

(28) $$\begin{array}{ccc} S_{1}\left(a_{1}\right) & = & r(H_{1}-B_{2}) \end{array}$$

(29) $$\begin{array}{ccc} S_{2}\left(a_{1}\right) & = & \frac{1}{\sqrt{2}}\left[r\left(B_{2}-H_{3}\right)+r\left(B_{2}-H_{4}\right)\right] \end{array}$$

(30) $$\begin{array}{ccc} S_{3}\left(a_{1}\right) & = & \frac{1}{\sqrt{2}}\left[∠\left(H_{1}-B_{2}-H_{3}\right)+∠\left(H_{1}-B_{2}-H_{4}\right)\right] \end{array}$$

(31) $$\begin{array}{ccc} S_{4}\left(b_{2}\right) & = & \frac{1}{\sqrt{2}}\left[r\left(B_{2}-H_{3}\right)-r\left(B_{2}-H_{4}\right)\right] \end{array}$$

(32) $$\begin{array}{ccc} S_{5}\left(b_{2}\right) & = & \frac{1}{\sqrt{2}}\left[∠\left(H_{1}-B_{2}-H_{3}\right)-∠\left(H_{1}-B_{2}-H_{4}\right)\right] \end{array}$$

(33) $$\begin{array}{ccc} S_{6}\left(b_{1}\right) & = & \mathrm{OUT}(H_{1}-B_{2}-H_{3}-H_{4}). \end{array}$$

As shown by the symmetry labels in Equations (28)–(33), borane is treated in $C_{2v}$ symmetry rather than its full $D_{3h}$ symmetry to simplify the coordinate system. The SIC coordinate systems utilized herein for BH${}_{3}$OH${}_{2}$ and BH${}_{2}$OH have been previously applied to the structurally similar AlH${}_{3}$OH${}_{2}$ [43] and AlH${}_{2}$OH [44] molecules, respectively. The total numbers of single-point energy computations to generate the QFFs for BH${}_{3}$OH${}_{2}$, BH${}_{2}$OH, BH${}_{3}$, and HBO are 19585, 3161, 413, and 55.

**Figure 5:**
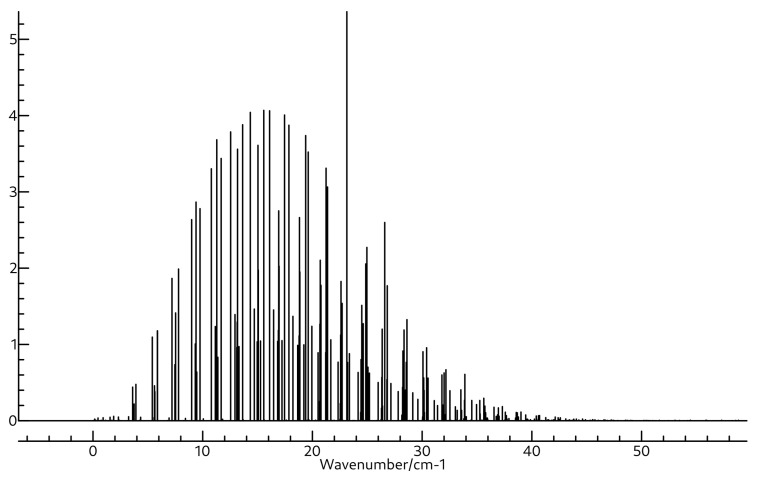
Simulated rotational spectrum of BH${}_{2}$OH using $A_{0}$, $B_{0}$, and $C_{0}$ shown in Table 3, and $\Delta_{J}$, $\Delta_{JK}$, $\Delta_{K}$, $\delta_{J}$, $\delta_{K}$, $\Phi_{K}$, $\Phi_{KJ}$, $\Phi_{JK}$, and $\Phi_{J}$ shown in Table S15 at a temperature of 94 K with Lorentzian line shapes with FWHMs of 0.015 cm${}^{-1}$.

**Figure 6:**
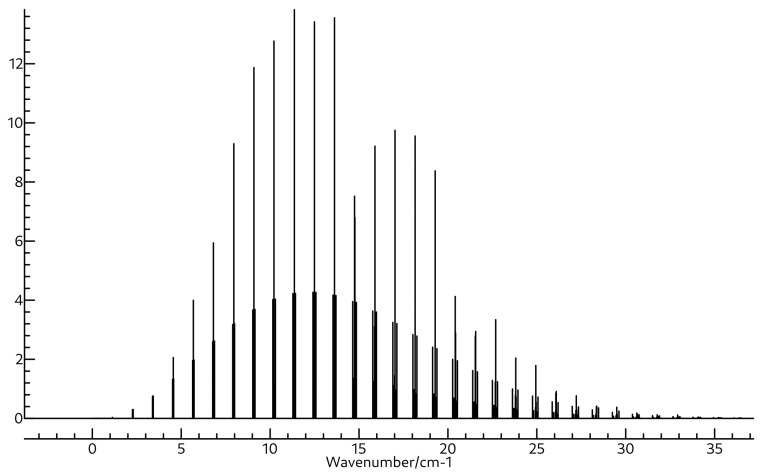
Simulated rotational spectrum of BH${}_{3}$OH${}_{2}$ using $A_{0}$, $B_{0}$, and $C_{0}$ shown in Table 4, and $\Delta_{J}$, $\Delta_{JK}$, $\Delta_{K}$, $\delta_{J}$, $\delta_{K}$, $\Phi_{K}$, $\Phi_{KJ}$, $\Phi_{JK}$, and $\Phi_{J}$ shown in Table S15 at a temperature of 94 K with Lorentzian line shapes with FWHMs of 0.015 cm${}^{-1}$.

**Figure 7:**
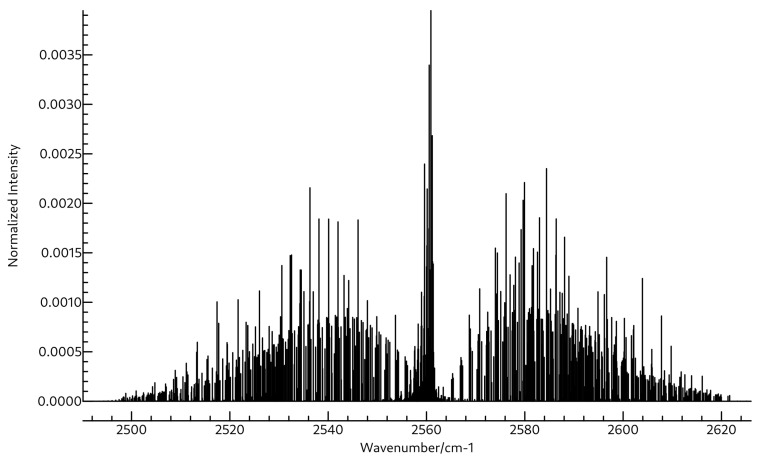
Simulated rovibrational spectrum of the first $\nu_{2}$ transition of BH${}_{2}$OH using $A_{0}$, $B_{0}$, $C_{0}$, $A_{2}$, $B_{2}$, and $C_{2}$ shown in Table 3, and $\Delta_{J}$, $\Delta_{JK}$, $\Delta_{K}$, $\delta_{J}$, $\delta_{K}$, $\Phi_{K}$, $\Phi_{KJ}$, $\Phi_{JK}$, and $\Phi_{J}$ shown in Table S15 at a temperature of 94 K with Lorentzian line shapes with FWHMs of 0.015 cm${}^{-1}$.

**Figure 8:**
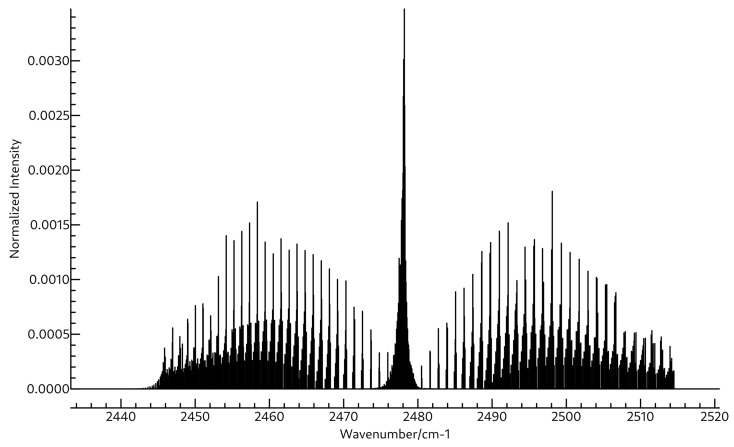
Simulated rovibrational spectrum of the first $\nu_{3}$ transition of BH${}_{3}$OH${}_{2}$ using $A_{0}$, $B_{0}$, $C_{0}$, $A_{2}$, $B_{2}$, and $C_{2}$ shown in Table 4, and $\Delta_{J}$, $\Delta_{JK}$, $\Delta_{K}$, $\delta_{J}$, $\delta_{K}$, $\Phi_{K}$, $\Phi_{KJ}$, $\Phi_{JK}$, and $\Phi_{J}$ shown in Table S15 at a temperature of 94 K with Lorentzian line shapes with FWHMs of 0.015 cm${}^{-1}$.

After the single-point energy computations for either QFF energy, the QFF function is fit using a least-squares procedure with sums of squared residuals on the order of 10${}^{-16}$ a.u.${}^{2}$ in all cases. The first fit yields the equilibrium geometries and a subsequent refitting zeroes the gradients and produces a new equilibrium geometry along with the corresponding force constants. These force constants are transformed from SICs to Cartesian coordinates using the INTDER program [45]. The Cartesian force constants are then utilized by the second-order rotational and vibrational perturbation theory [46] implementations in the SPECTRO software package [47] to generate the rovibrational spectral data [48,49]. Type 1 and 2 Fermi resonances, Fermi polyads [50], Coriolis resonances, and Darling-Dennison resonances are taken into account to further increase the accuracy of the rovibrational data [50,51]. The Fermi resonances are listed in the Supplementary Information (SI) in Tables S4, S6, S8, S10, S12, S14, S24, S28, S34, S40, S47 and S54. Additionally, the rotational constants from the SPECTRO program are used in the PGOPHER software package [52] to simulate the rotational and rovibrational spectra for BH${}_{2}$OH and BH${}_{3}$OH${}_{2}$ shown in Figures 5–8.

## 3. Results and Discussion

### 3.1. Benchmarks

As shown in Table 1 and Table 2, the F12-TZ-cCR QFF fundamental frequencies demonstrate excellent agreement with the available gas-phase experimental data. In the case of borane, the largest difference occurs in $\nu_{3}$ with a deviation of only 1.9 cm${}^{-1}$, and the mean absolute error (MAE) or unsigned averaged deviation across the three modes is 1.1 cm${}^{-1}$. This is in contrast to the F12-TZ results, which are a bit farther from the experimental values. Again for borane, the biggest deviation from experiment in the F12-TZ results is 7.5 cm${}^{-1}$ with an MAE of 3.8 cm${}^{-1}$. The two are more comparable for HBO, where F12-TZ achieves a respectable MAE of 3.9 cm${}^{-1}$ relative to the two available experimental frequencies. However, F12-TZ-cCR still has the slight edge with an MAE of 2.8 cm${}^{-1}$. Such performance indicates that both F12-TZ and F12-TZ-cCR can adequately handle the vibrational spectra of these two molecules, but for a slight increase (3484 versus 5397 seconds of wall time for BH${}_{3}$) in computational cost, F12-TZ-cCR provides a substantial increase in accuracy.

The same is true upon examination of the principal rotational constants. While F12-TZ-cCR exhibits fairly large deviations from the experimental data for borane giving an overall MAE of 218.3 MHz, F12-TZ performs much worse with an MAE of 750.2 MHz. This is in line with previous work on both F12-TZ [22,23,24] and F12-TZ-cCR [28], which demonstrates that accounting for the effects of core correlation in F12-TZ-cCR is necessary for producing more accurate rotational constants. In the present case, neither of the methodologies utilized herein seems to be achieving real accuracy, but F12-TZ-cCR still has a clear advantage. For HBO, that advantage becomes even more pronounced. Whereas F12-TZ has an MAE from the reported $B_{e}$ and $B_{0}$ values of 200.2 MHz, F12-TZ-cCR achieves a much more reasonable MAE of only 12.3 MHz. Looking at the vibrationally-averaged $B_{0}$ value tells the same story; the F12-TZ QFF differs from the experimental value by 197.9 MHz and the F12-TZ-cCR value by only 14.4 MHz. Differently, both F12-TZ and F12-TZ-cCR seem to capture the quartic and sextic distortion coefficients in the Watson S-reduced Hamiltonian presented at the bottom of Table 2. The two computational data sets agree very closely with each other in this case. Neither is more than 3 MHz away in the *D* constants (−32.746 compared to −29.834 MHz for $D_{JK}$) or 3 kHz in the *H* constants (17.898 compared to 15.047 kHz for $H_{KJ}$) from the available borane experimental values, and most of the differences are even smaller.

Finally, but perhaps most promisingly, the same trend is clear in the borinic acid data shown in Table 3. Compared to the available experimental vibrationally-averaged rotational constants, F12-TZ has an MAE difference of 308.7 MHz. In stark contrast, F12-TZ-cCR manages an MAE of only 22.3 MHz, and most of this is concentrated in the difference from $A_{0}$ of 66.0 MHz. The F12-TZ-cCR values for both $B_{0}$ and $C_{0}$ agree to within just over 0.5 MHz. Such exceptional agreement even exceeds the expected performance of F12-TZ-cCR, which previously achieved an average agreement of roughly 7.5 MHz on similar $B_{0}$ and $C_{0}$ rotational constants [28]. This suggests that F12-TZ-cCR is very well suited to the determination of the vibrational spectrum of borinic acid and for the elucidation of both the rotational and vibrational spectra of water borane. The accuracy of the rotational constants in the case of water borane is particularly important given its massive dipole moment of 4.24 D, which should make its rotational spectrum easier to obtain if the molecule itself can be isolated experimentally.

### 3.2. Spectroscopic Data

In light of the performance of the F12-TZ-cCR QFFs on HBO, borane, and the rotational constants of borinic acid, as well as the lack of experimental data on the vibrational frequencies of borinic acid and water borane, the F12-TZ-cCR fundamental frequencies reported herein are the most accurate values available for these two molecules. In some cases, such as the high-frequency O-H stretches, the good agreement between F12-TZ and F12-TZ-cCR adds further support to the theoretical quantification of these frequencies. Across BH${}_{3}$OH${}_{2}$ and BH${}_{2}$OH, the deviations in these frequencies are all less than 4 cm${}^{-1}$ with the largest difference occurring in $\nu_{1}$ of BH${}_{3}$OH${}_{2}$ at 3.9 cm${}^{-1}$. However, the B-H stretches are less consistent between the levels of theory. The $\nu_{3}$ antisymmetric B-H stretch of BH${}_{3}$OH${}_{2}$, for example, exhibits a difference of 12.1 cm${}^{-1}$ between F12-TZ and F12-TZ-cCR. The agreement is better across the board for BH${}_{2}$OH, but the $\nu_{2}$ antisymmetric B-H stretch still exhibits a difference of 6.0 cm${}^{-1}$ between the two treatments. Properly handling this mode is particularly important in light of its high intensity for both molecules. In both cases, the antisymmetric B-H stretch is the most intense mode, with that of BH${}_{2}$OH having an intensity of 169 km mol${}^{-1}$ and that of BH${}_{3}$OH${}_{2}$ even more intense at 206 km mol${}^{-1}$.

For BH${}_{2}$OH, the $\nu_{4}$ B-O stretch at 1352.8 cm${}^{-1}$ is the next most intense mode with a value of 152 km mol${}^{-1}$. The additional hydrogens in BH${}_{3}$OH${}_{2}$ damp both the frequency and intensity of the B-O stretch, decreasing the frequency to 399.4 cm${}^{-1}$ and the intensity to 82 km mol${}^{-1}$ in $\nu_{14}$. This frequency is considerably lower than the previously computed value for the B-N stretch in ammonia borane at 644 cm${}^{-1}$, but the intensity is much greater than the 12 km mol${}^{-1}$ reported therein [13]. Such a shift suggests that the B-O bond in BH${}_{3}$OH${}_{2}$ is actually weaker than the B-N bond in ammonia borane, and this is corroborated by the slightly longer 1.74485 Å B-O bond length compared to the 1.67308 Å B-N bond. These expected trends in the bond strengths are supported by F12-TZ bond strength computations, which give a value of −9.7 kcal mol${}^{-1}$ for the B-O bond and −25.7 kcal mol${}^{-1}$ for the B-N bond. In contrast, the B-H bonds of BH${}_{3}$OH${}_{2}$ are slightly shorter at 1.20516 Å compared to the 1.21685 Å observed for ammonia borane, and this is again consistent with the higher frequency B-H stretches observed in the present work.

Examining the rest of the anharmonic infrared intensities for BH${}_{2}$OH reveals that all but the $\nu_{5}$ B-O-H bend have intensities greater than 50 km mol${}^{-1}$, and even $\nu_{5}$ itself still has an intensity of 22 km mol${}^{-1}$. BH${}_{3}$OH${}_{2}$, on the other hand, has more low intensity fundamental vibrational frequencies, such as $\nu_{7}$, $\nu_{8}$, $\nu_{10}$, and $\nu_{12}$ at 12, 23, 13, and 1 km mol${}^{-1}$, respectively, but also more high intensity frequencies. Chief among these are the aforementioned antisymmetric B-H stretch of $\nu_{3}$, as well as the $\nu_{1}$ antisymmetric O-H stretch with an intensity of 133 km mol${}^{-1}$, the $\nu_{4}$ symmetric B-H stretch at 135 km mol${}^{-1}$, and the $\nu_{13}$ symmetric B-O-H bend at 179 km mol${}^{-1}$. For both molecules, the presence of these high intensity frequencies should help to facilitate their vibrational observation, if the molecules can be experimentally isolated in the gas phase. Further, the fact that there is little overlap between the frequencies of the most intense fundamentals means that the two can likely be disentangled if they are observed together in the same experiment.

The same is true for the rotational spectra of the two molecules also shown in Table 3 and Table 4. Whereas the $A_{0}$ constant for BH${}_{2}$OH is close to 172 GHz, that for BH${}_{3}$OH${}_{2}$ is much lower, near 87 GHz. Both molecules are near-prolate with κ values of −0.94 and −0.99 for BH${}_{2}$OH and BH${}_{3}$OH${}_{2}$, respectively. Clearly BH${}_{3}$OH${}_{2}$ is much closer to prolate, as evidenced by the mere 452.6 MHz separation between its $B_{0}$ and $C_{0}$ constants, which are found at 17,269.0 and 16,816.4 MHz. Again, these are quite far away from those of BH${}_{2}$OH, which are found experimentally at 30,470.22 and 25,812.73 MHz, suggesting that the two should be readily distinguished if observed in the same experiment. The quartic and sextic distortion coefficients for these two molecules are shown in Table S15 of the SI. The microwave spectra for BH${}_{2}$OH and BH${}_{3}$OH${}_{2}$ are shown in Figure 5 and Figure 6, respectively, and the rovibrational spectra for $\nu_{2}$ of BH${}_{2}$OH and $\nu_{3}$ of BH${}_{3}$OH${}_{2}$ are shown in Figure 7 and Figure 8.

### 3.3. ${}^{10}$B Isotopologues

All of the data discussed above are for the ${}^{11}$B isotope. Given the relatively high isotopic abundance of ${}^{10}$B, the computations are repeated for these isotopologues, and the results are reported in Tables S16–S55 of the SI. Overall, the trends are much the same as those observed for the ${}^{11}$B variants. Namely, the F12-TZ-cCR fundamental frequencies are all within 2 cm${}^{-1}$ of the reported gas-phase values for borane [29] and HBO [30]; and the MAE for the principal rotational constants of borane is comparable to that for the ${}^{11}$B variant at 252.3 MHz. The difference in the $B_{0}$ rotational constant of HBO is 15.1 MHz. However, the MAE for the vibrationally-averaged principal rotational constants of borinic acid is quite a bit higher than that of the ${}^{11}$B isotopologue at 312.4 MHz. Most of the deviation is again in the $A_{0}$ constant, but this time even $B_{0}$ and $C_{0}$ have deviations over 100 MHz. Regardless, the good general agreement with the available experimental results suggests that the F12-TZ-cCR spectral data reported herein for ${}^{10}$BH${}_{3}$OH${}_{2}$ and ${}^{10}$BH${}_{2}$OH should be reliable as well.

## 4. Conclusions

This work presents the most accurate rovibrational spectroscopic data for water borane, BH${}_{3}$OH${}_{2}$, and borinic acid, BH${}_{2}$OH, currently available. The existence of gas-phase vibrational and rotational experimental data for the related borane, BH${}_{3}$, and HBO molecules, as well as the vibrationally-averaged principal rotational constants of borinic acid, provide a wealth of benchmarking data for assessing the accuracy of the theoretical methods utilized for generating these novel data. In particular the recently developed F12-TZ-cCR QFF methodology performs substantially better than the more conventional F12-TZ methodology, which neglects explicit accounting for the effects of core correlation and scalar relativity. Both BH${}_{3}$OH${}_{2}$ and BH${}_{2}$OH have several vibrational frequencies each with intensities over 100 km mol${}^{-1}$, suggesting that these molecules will be readily observable in the infrared, if they can be experimentally isolated. Further, despite their structural similarity, these intense modes are sufficiently resolved that it should be possible to separate the two spectra if both molecules are produced in a single experiment. For BH${}_{2}$OH, the most intense modes occur at 2561.6 ($\nu_{2}$), 1352.8 ($\nu_{4}$), and 1171.1 ($\nu_{6}$) cm${}^{-1}$, while those for BH${}_{3}$OH${}_{2}$ are found at 3704.8 ($\nu_{1}$), 2488.4 ($\nu_{3}$), 2452.3 ($\nu_{4}$), 1176.8 ($\nu_{9}$), and 528.4 ($\nu_{13}$) cm${}^{-1}$. In terms of rotational spectra, BH${}_{2}$OH possesses a substantial dipole moment of 1.51 D that helped to facilitate its previous experimental detection by microwave.png spectroscopy. Likewise, BH${}_{3}$OH${}_{2}$ has an enormous dipole moment of 4.24 D. As a result, it should be readily rotationally detectable as well, again if it can be isolated in the laboratory. Such isolation is of substantial importance due to the potential for both BH${}_{3}$OH${}_{2}$ and BH${}_{2}$OH to be involved in the production of H${}_{2}$ from water, which could be a promising route to clean alternative fuels. Regardless of the application, the highly-accurate theoretical rovibrational spectral data presented herein will help to guide future experimental investigations toward the detection of these molecules.

## Figures and Tables

**Table 1:** Harmonic and anharmonic vibrational frequencies (in cm${}^{-1}$), MP2/aug-cc-pVTZ harmonic and anharmonic infrared intensities (in km mol${}^{-1}$, labeled *f*), equilibrium, vibrationally averaged, and singly-vibrationally excited principal rotational constants (in MHz), and dipole (in D) for HBO. Descriptions of the vibrational frequencies are given as linear combinations of SICs and as qualitative descriptions along with the symmetries.

	SICs	Description	Symmetry	*f*	F12-TZ	F12-TZ-cCR	Expt. ${}^{a}$
$\omega_{1}$	0.932$S_{2}$ − 0.068$S_{1}$	B-H stretch	σ	4	2890.3	2897.6	
$\omega_{2}$	0.932$S_{1}$ + 0.068$S_{2}$	B-O stretch	σ	35	1837.8	1845.8	
$\omega_{3}$	0.500$S_{3}$ + 0.500$S_{4}$	H-B-O bend	π	12	764.6	764.9	
ZPVE					3098.4	3111.3	
$\nu_{1}$	0.932$S_{2}$ − 0.068$S_{1}$	B-H stretch	σ	4	2779.5	2795.2	
$\nu_{2}$	0.932$S_{1}$ + 0.068$S_{2}$	B-O stretch	σ	33	1822.8	1829.5	1825.5610
$\nu_{3}$	0.500$S_{3}$ + 0.500$S_{4}$	H-B-O bend	π	11	749.4	756.1	754.4163
$B_{e}$					39,198.2	39,411.0	39,400.668
$B_{0}$					39,026.3	39,238.6	39,224.247
$B_{1}$					38,757.3	38,967.4	
$B_{2}$					38,767.3	38,978.0	
$B_{3}$					39,118.3	39,331.9	
μ					2.74		

${}^{a}$ From Ref. [30].

**Table 2:** Harmonic and anharmonic vibrational frequencies (in cm${}^{-1}$), MP2/aug-cc-pVTZ harmonic and anharmonic infrared intensities (in km mol${}^{-1}$, labeled *f*), equilibrium, vibrationally averaged, and singly-vibrationally excited principal rotational constants (in MHz), and dipole (in D) for BH${}_{3}$. Descriptions of the vibrational frequencies are given as linear combinations of SICs and as qualitative descriptions along with the symmetries.

	SICs	Description	Symmetry	*f*	F12-TZ	F12-TZ-cCR	Expt. ${}^{a}$
$\omega_{1}$	0.334$S_{1}$ − 0.167$S_{2}$+ 0.501$S_{4}$	antisymm. stretch	$b_{2}$	133	2700.3	2708.5	
$\omega_{2}$	0.667$S_{2}$ + 0.333$S_{1}$	symm. stretch	$a_{1}$	0	2567.6	2575.0	
$\omega_{3}$	0.501$S_{5}$ + 0.501$S_{3}$	in-plane rock	$b_{2}$	17	1218.4	1221.4	
$\omega_{4}$	1.000$S_{6}$	out-of-plane wag	$b_{1}$	90	1156.9	1159.9	
ZPVE					5724.1	5740.6	
$\nu_{1}$	0.334$S_{1}$ − 0.167$S_{2}$ + 0.501$S_{4}$	antisymm. stretch	$b_{2}$	135	2594.0	2601.9	2601.5779
$\nu_{2}$	0.667$S_{2}$ + 0.333$S_{1}$	symm. stretch	$a_{1}$	0	2498.4	2505.8	
$\nu_{3}$	0.501$S_{5}$ + 0.501$S_{3}$	in-plane rock	$b_{2}$	17	1194.9	1197.9	1196.0217
$\nu_{4}$	1.000$S_{6}$	out-of-plane wag	$b_{1}$	86	1144.9	1148.7	1147.49087
$B_{e}$					236,255.9	237,314.5	
$C_{e}$					118,127.8	118,657.3	
$B_{0}$					235,210.3	236,268.5	236,071.4
$C_{0}$					115,831.7	116,349.1	116,283.5
$B_{1}$					233,018.6	234,064.7	234,104.0
$C_{1}$					114,910.4	115,422.2	115,411.0
$B_{2}$					233,201.4	234,248.8	
$C_{2}$					114,827.3	115,339.3	
$B_{3}$					238,431.6	239,513.3	23,9112.4
$C_{3}$					114,665.5	115,177.1	11,5130.0
$B_{4}$					233,068.4	234,113.6	23,4801.4
$C_{4}$					116,418.6	116,940.3	116,643.1
$D_{J}$					17.016	17.160	18.199
$D_{JK}$					−29.579	−29.834	−32.746
$D_{K}$					13.676	13.795	15.46
$H_{J}\times 10^{3}$					3.476	3.521	3.639
$H_{JK}\times 10^{3}$					−12.606	−12.771	−14.48
$H_{KJ}\times 10^{3}$					14.853	15.047	17.898
$H_{K}\times 10^{3}$					−5.711	−5.786	−7.081
μ					0.00		

${}^{a}$ From Ref. [29].

**Table 3:** Harmonic and anharmonic vibrational frequencies (in cm${}^{-1}$), MP2/aug-cc-pVTZ harmonic and anharmonic infrared intensities (in km mol${}^{-1}$, labeled *f*), equilibrium, vibrationally averaged, and singly-vibrationally excited principal rotational constants (in MHz), and dipole (in D) for BH${}_{2}$OH. Descriptions of the vibrational frequencies are given as linear combinations of SICs and as qualitative descriptions along with the symmetries.

	SICs	Description	Symmetry	*f*	F12-TZ	F12-TZ-cCR	Expt. ${}^{a}$
$\omega_{1}$	1.000$S_{1}$	O-H stretch	$a^{\prime }$	83	3867.8	3869.8	
$\omega_{2}$	0.739$S_{3}$ − 0.260$S_{4}$	B-H antisymm. stretch	$a^{\prime \prime }$	172	2673.2	2679.9	
$\omega_{3}$	0.739$S_{4}$ + 0.259$S_{3}$	B-H symm. stretch	$a^{\prime }$	104	2572.8	2579.4	
$\omega_{4}$	0.568$S_{2}$ − 0.247$S_{7}$ − 0.182$S_{6}$	B-O stretch	$a^{\prime }$	151	1376.7	1382.0	
$\omega_{5}$	0.577$S_{5}$ − 0.273$S_{7}$ + 0.099$S_{6}$ − 0.050$S_{2}$	H-O-B bend	$a^{\prime }$	6	1190.9	1194.0	
$\omega_{6}$	0.412$S_{6}$ + 0.380$S_{2}$ + 0.167$S_{7}$	H-B-O bend	$a^{\prime }$	112	1188.2	1192.4	
$\omega_{7}$	0.559$S_{9}$ − 0.441$S_{8}$	out-of-plane wag	$a^{\prime \prime }$	51	1061.9	1065.1	
$\omega_{8}$	0.381$S_{5}$ + 0.314$S_{7}$ − 0.308$S_{6}$	in-plane rock	$a^{\prime }$	57	894.0	897.8	
$\omega_{9}$	0.559$S_{8}$ + 0.441$S_{9}$	torsion	$a^{\prime \prime }$	83	785.4	789.3	
ZPVE					7708.4	7725.5	
$\nu_{1}$	1.000$S_{1}$	O-H stretch	$a^{\prime }$	77	3681.1	3683.0	
$\nu_{2}$	0.739$S_{3}$ − 0.260$S_{4}$	B-H antisymm. stretch	$a^{\prime \prime }$	169	2555.6	2561.6	
$\nu_{3}$	0.739$S_{4}$ + 0.259$S_{3}$	B-H symm. stretch	$a^{\prime }$	76	2456.2	2462.4	
$\nu_{4}$	0.568$S_{2}$ − 0.247$S_{7}$ − 0.182$S_{6}$	B-O stretch	$a^{\prime }$	152	1347.7	1352.8	
$\nu_{5}$	0.577$S_{5}$ − 0.273$S_{7}$ + 0.099$S_{6}$ − 0.050$S_{2}$	H-O-B bend	$a^{\prime }$	22	1154.1	1158.3	
$\nu_{6}$	0.412$S_{6}$ + 0.380$S_{2}$ + 0.167$S_{7}$	H-B-O bend	$a^{\prime }$	98	1167.8	1171.1	
$\nu_{7}$	0.559$S_{9}$ − 0.441$S_{8}$	out-of-plane wag	$a^{\prime \prime }$	50	1048.1	1051.4	
$\nu_{8}$	0.381$S_{5}$ + 0.314$S_{7}$ − 0.308$S_{6}$	in-plane rock	$a^{\prime }$	59	880.7	882.8	
$\nu_{9}$	0.559$S_{8}$ + 0.441$S_{9}$	torsion	$a^{\prime \prime }$	82	753.5	751.0	
$A_{e}$					172,890.0	173,612.5	
$B_{e}$					30,552.4	30,704.1	
$C_{e}$					25,964.1	26,090.0	
$A_{0}$					171,969.4	172,687.1	17,2621.1
$B_{0}$					30,319.5	30,469.7	30,470.22
$C_{0}$					25,689.0	25,813.1	25,812.73
$A_{1}$					170,127.7	170,835.2	
$B_{1}$					30,294.4	30,444.4	
$C_{1}$					25,631.6	25,755.3	
$A_{2}$					170,798.0	171,510.7	
$B_{2}$					30,278.4	30,428.3	
$C_{2}$					25,646.5	25,770.4	
$A_{3}$					170,001.1	170,709.4	
$B_{3}$					30,306.0	30,456.1	
$C_{3}$					25,638.0	25,761.7	
$A_{4}$					172,528.1	173,243.9	
$B_{4}$					30,328.9	30,477.1	
$C_{4}$					25,515.1	25,637.8	
$A_{5}$					175,303.8	176,002.0	
$B_{5}$					30,366.7	30,511.9	
$C_{5}$					25,594.4	25,712.3	
$A_{6}$					172,698.2	173,646.6	
$B_{6}$					30,278.2	30,437.8	
$C_{6}$					25,601.6	25,730.7	
$A_{7}$					170,201.5	170,730.6	
$B_{7}$					30,086.2	30,232.3	
$C_{7}$					25,724.7	25,849.0	
$A_{8}$					176,655.6	177,443.9	
$B_{8}$					30,274.3	30,423.6	
$C_{8}$					25,610.4	25,733.9	
$A_{9}$					167,569.6	168,210.5	
$B_{9}$					30,196.4	30,346.5	
$C_{9}$					25,689.4	25,813.5	
μ					1.51		1.506

${}^{a}$ From Ref. [31].

**Table 4:** Harmonic and anharmonic vibrational frequencies (in cm${}^{-1}$), MP2/aug-cc-pVTZ harmonic and anharmonic infrared intensities (in km mol${}^{-1}$, labeled *f*), equilibrium, vibrationally averaged, and singly-vibrationally excited principal rotational constants (in MHz), and dipole (in D) for BH${}_{3}$OH${}_{2}$. Descriptions of the vibrational frequencies are given as linear combinations of SICs and as qualitative descriptions along with the symmetries.

	SICs	Description	Symmetry	*f*	F12-TZ	F12-TZ-cCR
$\omega_{1}$	1.000$S_{11}$	antisymm. O-H stretch	$a^{\prime \prime }$	151	3889.9	3892.5
$\omega_{2}$	1.001$S_{4}$	symm. O-H stretch	$a^{\prime }$	56	3789.2	3792.0
$\omega_{3}$	1.002$S_{10}$	antisymm. B-H stretch	$a^{\prime \prime }$	215	2586.8	2593.6
$\omega_{4}$	0.623$S_{3}$ − 0.378$S_{1}$	symm. B-H stretch	$a^{\prime }$	218	2556.1	2562.9
$\omega_{5}$	0.624$S_{1}$ + 0.378$S_{3}$	B-H breathing	$a^{\prime }$	55	2482.1	2488.7
$\omega_{6}$	0.723$S_{8}$ − 0.285$S_{7}$	H-O-H bend	$a^{\prime }$	92	1653.8	1655.0
$\omega_{7}$	0.663$S_{14}$ − 0.305$S_{15}$	B-H in-plane rock	$a^{\prime \prime }$	13	1208.4	1211.7
$\omega_{8}$	0.574$S_{9}$ + 0.278$S_{5}$ + 0.181$S_{6}$	B-H in-plane bend	$a^{\prime }$	29	1200.4	1204.1
$\omega_{9}$	0.462$S_{6}$ − 0.438$S_{9}$ + 0.145$S_{5}$	B-H out-of-plane wag	$a^{\prime }$	131	1194.6	1198.3
$\omega_{10}$	0.670$S_{12}$ − 0.320$S_{13}$	torsion	$a^{\prime \prime }$	13	1025.9	1031.4
$\omega_{11}$	0.503$S_{5}$ − 0.344$S_{6}$ − 0.098$S_{7}$ − 0.057$S_{9}$	O-H wag	$a^{\prime }$	56	952.3	957.4
$\omega_{12}$	0.675$S_{13}$ + 0.296$S_{12}$	antisymm. B-O-H bend	$a^{\prime \prime }$	3	647.3	651.4
$\omega_{13}$	0.629$S_{7}$ + 0.311$S_{8}$ + 0.083$S_{5}$ + 0.057$S_{9}$	symm. B-O-H bend	$a^{\prime }$	177	607.2	610.4
$\omega_{14}$	1.037$S_{2}$	B-O stretch	$a^{\prime }$	84	467.0	472.1
$\omega_{15}$	0.695$S_{15}$ + 0.299$S_{14}$	torsion	$a^{\prime \prime }$	39	156.4	158.1
ZPVE					11,981.3	12,050.8
$\nu_{1}$	1.000$S_{11}$	antisymm. O-H stretch	$a^{\prime \prime }$	133	3700.9	3704.8
$\nu_{2}$	1.001$S_{4}$	symm. O-H stretch	$a^{\prime }$	48	3612.6	3615.6
$\nu_{3}$	1.002$S_{10}$	antisymm. B-H stretch	$a^{\prime \prime }$	206	2476.3	2488.4
$\nu_{4}$	0.623$S_{3}$ − 0.378$S_{1}$	symm. B-H stretch	$a^{\prime }$	135	2444.9	2452.3
$\nu_{5}$	0.624$S_{1}$ + 0.378$S_{3}$	B-H breathing	$a^{\prime }$	94	2433.5	2441.2
$\nu_{6}$	0.723$S_{8}$ − 0.285$S_{7}$	H-O-H bend	$a^{\prime }$	60	1627.8	1634.3
$\nu_{7}$	0.663$S_{14}$ − 0.305$S_{15}$	B-H in-plane rock	$a^{\prime \prime }$	12	1178.2	1179.4
$\nu_{8}$	0.574$S_{9}$ + 0.278$S_{5}$ + 0.181$S_{6}$	B-H in-plane bend	$a^{\prime }$	23	1172.3	1175.5
$\nu_{9}$	0.462$S_{6}$ − 0.438$S_{9}$ + 0.145$S_{5}$	B-H out-of-plane wag	$a^{\prime }$	139	1169.0	1176.8
$\nu_{10}$	0.670$S_{12}$ − 0.320$S_{13}$	torsion	$a^{\prime \prime }$	13	944.9	971.9
$\nu_{11}$	0.503$S_{5}$ − 0.344$S_{6}$ − 0.098$S_{7}$ − 0.057$S_{9}$	O-H wag	$a^{\prime }$	34	898.9	927.2
$\nu_{12}$	0.675$S_{13}$ + 0.296$S_{12}$	antisymm. B-O-H bend	$a^{\prime \prime }$	1	609.4	619.4
$\nu_{13}$	0.629$S_{7}$ + 0.311$S_{8}$ + 0.083$S_{5}$ + 0.057$S_{9}$	symm. B-O-H bend	$a^{\prime }$	179	543.9	528.4
$\nu_{14}$	1.037$S_{2}$	B-O stretch	$a^{\prime }$	82	397.1	399.4
$\nu_{15}$	0.695$S_{15}$ + 0.299$S_{14}$	torsion	$a^{\prime \prime }$	35	92.1	241.2
$A_{e}$					87,262.1	87,643.7
$B_{e}$					17,819.1	17,935.7
$C_{e}$					17,330.7	17,440.4
$A_{0}$					86,509.6	86,912.3
$B_{0}$					17,143.2	17,269.0
$C_{0}$					16,699.3	16,816.4
$A_{1}$					86,052.2	86,451.9
$B_{1}$					17,149.6	17,274.8
$C_{1}$					16,716.3	16,832.9
$A_{2}$					85,973.9	86,373.0
$B_{2}$					17,147.4	17,272.8
$C_{2}$					16,701.5	16,818.2
$A_{3}$					86,275.9	86,681.7
$B_{3}$					17,212.6	17,338.0
$C_{3}$					16,762.2	16,878.9
$A_{4}$					85,874.1	86,271.5
$B_{4}$					17,229.3	17,354.6
$C_{4}$					16,768.6	16,885.2
$A_{5}$					85,926.2	86,325.9
$B_{5}$					17,193.6	17,319.1
$C_{5}$					16,754.8	16,871.6
$A_{6}$					86,341.9	86,745.4
$B_{6}$					17,166.0	17,292.6
$C_{6}$					16,679.8	16,796.9
$A_{7}$					88,456.0	89,000.3
$B_{7}$					17,140.2	17,265.6
$C_{7}$					16,727.6	16,845.1
$A_{8}$					84,427.7	84,908.1
$B_{8}$					17,124.3	17,239.7
$C_{8}$					16,675.4	16,786.5
$A_{9}$					85,882.3	86,067.2
$B_{9}$					17,111.5	17,248.4
$C_{9}$					16,649.4	16,759.4
$A_{10}$					87,344.8	87,759.8
$B_{10}$					16,917.6	17,046.2
$C_{10}$					16,453.6	16,586.5
$A_{11}$					86,365.5	86,750.3
$B_{11}$					16,885.5	17,012.9
$C_{11}$					16,485.5	16,604.8
$A_{12}$					87,566.4	87,974.7
$B_{12}$					16,989.2	17,117.7
$C_{12}$					16,514.9	16,634.0
$A_{13}$					86,943.7	87,346.7
$B_{13}$					16,960.9	17,088.5
$C_{13}$					16,516.0	16,634.8
$A_{14}$					86,280.7	86,678.2
$B_{14}$					16,619.7	16,744.1
$C_{14}$					16,201.6	16,317.7
$A_{15}$					86,427.9	86,887.0
$B_{15}$					16,951.3	17,088.7
$C_{15}$					16,616.6	16,743.7
μ					4.24	

## Data Availability

All of the data supporting the conclusions presented in this work are available within the body of the text and in the Supplementary Information.

## Supplementary Materials

The following are available online, Tables S1–S14: Geometrical Parameters and Fermi Resonances for the ${}^{11}$B Isotopologues, Table S15: Quartic and Sextic Distortion Coefficients for BH${}_{2}$OH and BH${}_{3}$OH${}_{2}$, Tables S16–S55: Rovibrational Spectral Data for the ${}^{10}$B Isotopologues, Tables S56–S58: Dipole Components.
