# Supplementary material for: Anharmonic Vibrational Frequencies of Water Borane and Associated Molecules
Source: Molecules. 2021 Dec 3;26(23):7348. doi: 10.3390/molecules26237348 (PMC8658819; doi:10.3390/molecules26237348)
Supplement: Supplementary file 1 [file molecules-26-07348-s001.zip › molecules-1461771-supplementary.pdf]

Supplementary Information for Anharmonic vibrational frequencies of water borane and associated molecules

Brent R. Westbrook and Ryan C. Fortenberry*

Department of Chemistry & Biochemistry, University of Mississippi, University, MS 38677-1848, USA

E-mail: r410@olemiss.edu

Geometry of HBO

Table S1: F12-TZ Geom (A or Deg)

COORD	R(EQUIL)	R(ALPHA)
r(O1-B2)	1.2039214	1.2079482
r(B2-H3)	1.1689410	1.1696399

Table S2: F12-TZ-cCR Geom (A or Deg)

COORD	R(EQUIL)	R(ALPHA)
r(O1-B2)	1.2004938	1.2045030
r(B2-H3)	1.1666100	1.1672980

Geometry and Fermi Resonances of BH_3

Table S3: F12-TZ Geom (A or Deg)

COORD	R(EQUIL)	R(ALPHA)
r(H1-B2)	1.1895401	1.2030555
r(B2-H3)	1.1895401	1.2030448
r(B2-H4)	1.1895401	1.2030448
∠(B2-H1-H3)	119.9999994	119.9997808
∠(B2-H1-H4)	119.9999994	119.9997808

Table S4: F12-TZ Fermi Resonances

$2\nu_4 = \nu_3$

Table S5: F12-TZ-cCR Geom (A or Deg)

COORD	R(EQUIL)	R(ALPHA)
r(H1-B2)	1.1868837	1.2003795
r(B2-H3)	1.1868837	1.2003684
r(B2-H4)	1.1868837	1.2003684
∠(B2-H1-H3)	119.9999998	119.9997914
∠(B2-H1-H4)	119.9999998	119.9997914

Table S6: F12-TZ-cCR Fermi Resonances

$2\nu_4 = \nu_3$

Geometry and Fermi Resonances of BH_2OH

Table S7: F12-TZ Geom (A or Deg)

COORD	R(EQUIL)	R(ALPHA)
r(H1-O2)	0.9590492	0.9600299
r(O2-B3)	1.3531711	1.3597759
r(B3-H4)	1.1910927	1.2019218
r(B3-H5)	1.1966740	1.2081124
∠(O2-H1-B3)	112.8640075	113.3294319
∠(B3-O2-H4)	116.7657426	116.8103763
∠(B3-O2-H5)	120.4030231	120.5585208

Table S8: F12-TZ Fermi Resonances

$$2\nu_4=\nu_5+\nu_4=\nu_6+\nu_4=\nu_2$$
$$2\nu_4=2\nu_5=2\nu_6=\nu_5+\nu_4=\nu_6+\nu_4=\nu_6+\nu_5=\nu_3$$
$$2\nu_9=\nu_4$$

Table S9: F12-TZ-cCR Geom (A or Deg)

COORD	R(EQUIL)	R(ALPHA)
r(H1-O2)	0.9582831	0.9593249
r(O2-B3)	1.3495206	1.3561060
r(B3-H4)	1.1886041	1.1994419
r(B3-H5)	1.1941314	1.2055777
∠(O2-H1-B3)	112.9175285	113.3845673
∠(B3-O2-H4)	116.7901636	116.8344451
∠(B3-O2-H5)	120.4253816	120.5827740

Table S10: F12-TZ-cCR Fermi Resonances

$2\nu_4$=ν_5+ν_4=ν_6+ν_4=ν_2
$2\nu_4$=$2\nu_5$=$2\nu_6$=ν_5+ν_4=ν_6+ν_4=ν_6+ν_5=ν_3
$2\nu_9$=ν_4

Geometry and Fermi Resonances of BH_3OH_2

Table S11: F12-TZ Geom (A or Deg)

COORD	R(EQUIL)	R(ALPHA)
r(H1-B2)	1.2057530	1.2122329
r(B2-O3)	1.7113675	1.7525810
r(B2-H4)	1.2001106	1.2054761
r(B2-H5)	1.2001106	1.2054761
r(O3-H6)	0.9623613	0.9439335
r(O3-H7)	0.9623613	0.9439335
∠(B2-H1-O3)	102.9894094	102.5393695
∠(B2-H5-O3)	101.3072577	100.9632343
∠(B2-H4-O3)	101.3072577	100.9632343
∠(O3-H6-B2)	108.3323147	109.1306116
∠(O3-H7-B2)	108.3323147	109.1306116

Table S12: F12-TZ Fermi Resonances

$$\nu_{14}+\nu_{12}=\nu_{15}+\nu_{11}=\nu_{10}$$
$$\nu_{15}+\nu_{12}=\nu_{11}$$
$$\nu_{15}+\nu_{13}=\nu_{12}$$
$$2\nu_{15}=\nu_{14}$$
$$\nu_{8}+\nu_{7}=\nu_{9}+\nu_{7}=\nu_{15}+\nu_{4}=\nu_{15}+\nu_{5}=\nu_{3}$$
$$2\nu_{7}=2\nu_{8}=2\nu_{9}=\nu_{9}+\nu_{8}=\nu_{4}$$
$$2\nu_{7}=2\nu_{8}=2\nu_{9}=\nu_{9}+\nu_{8}=\nu_{5}$$
$$\nu_{12}+\nu_{10}=\nu_{13}+\nu_{11}=\nu_{6}$$
$$\nu_{13}+\nu_{12}=\nu_{14}+\nu_{12}=\nu_{15}+\nu_{11}=\nu_{7}$$
$$2\nu_{12}=2\nu_{13}=\nu_{8}$$
$$2\nu_{12}=2\nu_{13}=\nu_{14}+\nu_{13}=\nu_{15}+\nu_{10}=\nu_{9}$$

Table S13: F12-TZ-cCR Geom (A or Deg)

COORD	R(EQUIL)	R(ALPHA)
r(H1-B2)	1.2031473	1.2096563
r(B2-O3)	1.7047509	1.7448492
r(B2-H4)	1.1975300	1.2029102
r(B2-H5)	1.1975300	1.2029102
r(O3-H6)	0.9615372	0.9433751
r(O3-H7)	0.9615372	0.9433751
∠(B2-H1-O3)	103.1117956	102.7076490
∠(B2-H5-O3)	101.4206664	101.0778517
∠(B2-H4-O3)	101.4206664	101.0778517
∠(O3-H6-B2)	108.3895485	109.2684532
∠(O3-H7-B2)	108.3895485	109.2684532

Table S14: F12-TZ-cCR Fermi Resonances

$\nu_{14}+\nu_{12}=\nu_{15}+\nu_{11}=\nu_{10}$

$\nu_{15}+\nu_{12}=\nu_{11}$

$\nu_{15}+\nu_{13}=\nu_{12}$

$2\nu_{15}=\nu_{14}$

$\nu_{8}+\nu_{7}=\nu_{9}+\nu_{7}=\nu_{15}+\nu_{4}=\nu_{15}+\nu_{5}=\nu_{3}$

$2\nu_{7}=2\nu_{8}=2\nu_{9}=\nu_{9}+\nu_{8}=\nu_{4}$

$2\nu_{7}=2\nu_{8}=2\nu_{9}=\nu_{9}+\nu_{8}=\nu_{5}$

$\nu_{12}+\nu_{10}=\nu_{13}+\nu_{11}=\nu_{6}$

$\nu_{13}+\nu_{12}=\nu_{14}+\nu_{12}=\nu_{15}+\nu_{11}=\nu_{7}$

$2\nu_{12}=2\nu_{13}=\nu_{8}$

$2\nu_{12}=2\nu_{13}=\nu_{14}+\nu_{13}=\nu_{15}+\nu_{10}=\nu_{9}$

Table S15: Quartic and sextic distortion coefficients for BH_2OH and BH_3OH_2

	BH_2OH			BH_3OH_2		
	Units	F12-TZ	F12-TZ-cCR	Units	F12-TZ	F12-TZ-cCR
Δ_J	kHz	62.886	63.311	kHz	93.662	93.362
Δ_K	MHz	5.396	5.443	kHz	440.009	444.770
Δ_{JK}	kHz	525.836	528.355	kHz	96.980	99.009
δ_J	kHz	10.383	10.464	kHz	2.948	2.961
δ_K	kHz	461.601	464.423	kHz	−544.716	−545.280
Φ_J	mHz	93.254	94.414	Hz	−1.240	−1.225
Φ_K	Hz	729.279	738.813	Hz	63.631	61.418
Φ_{JK}	Hz	10.518	10.593	Hz	14.849	14.171
Φ_{KJ}	Hz	−18.973	−19.167	Hz	−57.772	−54.648
ϕ_j	mHz	49.380	49.939	mHz	−42.333	−42.306
ϕ_{jk}	Hz	6.087	6.132	Hz	16.572	13.462
ϕ_k	Hz	291.946	294.132	kHz	1.923	1.765

F12-TZ spectral data of $H^{10}BO$

Table S16: Freqs, ZPT=3139.0 (cm-1)

Mode	HARM	FUND	CORR
1	2914.9	2802.4	2802.4
2	1876.5	1861.7	1861.7
3	774.1	758.4	758.4

Table S17: ABC (MHz)

Constant	Value
B_e	40551.4
B_0	40371.0
B_1	40071.2
B_2	40102.2
B_3	40474.9

Table S18: Geom (A or Deg)

COORD	R(EQUIL)	R(ALPHA)
r(O1-B2)	1.2039214	1.2079960
r(B2-H3)	1.1689410	1.1698382

F12-TZ-cCR spectral data of $H^{10}BO$

Table S19: Freqs, ZPT=3152.2 (cm-1)

Mode	HARM	FUND	CORR	Expt[a]
1	2922.4	2818.8	2818.8	
2	1884.6	1868.2	1868.2	1863.56
3	774.4	765.3	765.3	763.6253

[a]From Ref. 30

Table S20: ABC (MHz)

Constant	Value	Expt[a]
B_e	40771.2	40759.482
B_0	40590.2	40575.395
B_1	40287.9	
B_2	40319.8	
B_3	40695.5	

[a]From Ref. 30

Table S21: Geom (A or Deg)

COORD	R(EQUIL)	R(ALPHA)
r(O1-B2)	1.2004938	1.2045502
r(B2-H3)	1.1666100	1.1674969

F12-TZ spectral data for $^{10}BH_3$

Table S22: Freqs, ZPT=5730.2 (cm-1)

Mode	HARM	FUND	CORR
1	2567.6	2476.5	2501.8
2	1169.2	1156.8	1156.8
3	2716.0	2608.1	2608.1
4	1224.3	1200.5	1200.5

Table S23: ABC (MHz)

Constant	Value
A_e	236255.9
B_e	118127.8
A_0	235197.2
C_0	115824.2
A_1	233188.4
C_1	114819.8
A_2	233036.3
C_2	116417.4
A_3	232974.2
C_3	114876.9
A_4	238446.4
C_4	114673.5

Table S24: Geom (A or Deg)

COORD	R(EQUIL)	R(ALPHA)
r(H1-B2)	1.1895401	1.2030977
r(B2-H3)	1.1895401	1.2030869
r(B2-H4)	1.1895401	1.2030869
∠(B2-H1-H3)	119.9999994	119.9997784
∠(B2-H1-H4)	119.9999994	119.9997784

Table S25: Fermi Resonances

$2\nu_4 = \nu_3$

F12-TZ-cCR spectral data for $^{10}BH_3$

Table S26: Freqs, ZPT=5746.7 (cm-1)

Mode	HARM	FUND	CORR	Expt[a]
1	2575.0	2483.9	2509.2	
2	1172.2	1160.6	1160.6	1159.4088
3	2724.3	2616.1	2616.1	2615.8045
4	1227.3	1203.4	1203.4	1201.8051

[a]From Ref. 29

Table S27: ABC (MHz)

Constant	Value	Expt[a]
A_e	237314.5	
C_e	118657.3	
A_0	236255.3	236063.9
C_0	116341.6	116283.5
A_1	234235.7	
C_1	115331.8	
A_2	234081.4	234614.9
C_2	116939.1	116580.6
A_3	234020.0	234037.8
C_3	115388.5	115369.4
A_4	239528.2	238820.4
C_4	115185.2	115317.6

[a]From Ref. 29

Table S28: Geom (A or Deg)

COORD	R(EQUIL)	R(ALPHA)
r(H1-B2)	1.1868837	1.2004216
r(B2-H3)	1.1868837	1.2004104
r(B2-H4)	1.1868837	1.2004104
∠(B2-H1-H3)	119.9999998	119.9997890
∠(B2-H1-H4)	119.9999998	119.9997890

Table S29: Fermi Resonances

$2\nu_4 = \nu_3$

F12-TZ spectral data of $^{10}BH_2OH$

Table S30: Freqs, ZPT=7750.1 (cm-1)

Mode	HARM	FUND	CORR
1	3867.9	3681.1	3681.1
2	2689.1	2570.3	2570.2
3	2579.1	2468.4	2466.5
4	1411.1	1380.9	1380.7
5	1198.8	1162.0	1162.0
6	1192.4	1171.9	1171.9
7	1075.8	1061.7	1061.7
8	896.8	883.1	883.1
9	785.5	753.5	753.5

Table S31: ABC (MHz)

Constant	Value	Expt[a]
A_e	172909.8	
B_e	31633.8	
C_e	26741.5	
A_0	171975.4	172629.4
B_0	31386.8	31542.88
C_0	26451.5	26578.55
A_1	170134.3	
B_1	31360.8	
C_1	26391.2	
A_2	170802.3	
B_2	31336.6	
C_2	26403.1	
A_3	169961.8	
B_3	31369.2	
C_3	26392.6	
A_4	172445.1	
B_4	31353.3	
C_4	26259.1	
A_5	175253.6	
B_5	31436.5	
C_5	26353.2	
A_6	172795.4	
B_6	31363.1	
C_6	26370.0	
A_7	170226.5	
B_7	31167.4	
C_7	26490.6	
A_8	176740.2	
B_8	31338.2	
C_8	26370.4	
A_9	167550.4	
B_9	31261.6	
C_9	26453.7	
K	-0.93217	

[a]From Ref. 31

Table S32: Deltas

Constant	Units	Value
Δ_J	kHz	67.169
Δ_K	MHz	5.384
Δ_{JK}	kHz	538.437
δ_J	kHz	11.493
δ_K	kHz	484.480

Table S33: Phis

Constant	Units	Value
Φ_J	mHz	115.708
Φ_K	Hz	732.806
Φ_{JK}	Hz	11.499
Φ_{KJ}	Hz	-22.222
ϕ_j	mHz	59.365
ϕ_{jk}	Hz	6.693
ϕ_k	Hz	302.544

Table S34: Geom (A or Deg)

COORD	R(EQUIL)	R(ALPHA)
r(H1-O2)	0.9590492	0.9600432
r(O2-B3)	1.3531711	1.3599148
r(B3-H4)	1.1910927	1.2020084
r(B3-H5)	1.1966740	1.2082132
∠(O2-H1-B3)	112.8640075	113.3286351
∠(B3-O2-H4)	116.7657426	116.8087292
∠(B3-O2-H5)	120.4030231	120.5575415

Table S35: Fermi Resonances

$2\nu_4 = \nu_5 + \nu_4 = \nu_6 + \nu_4 = \nu_2$

$2\nu_5 = 2\nu_6 = \nu_5 + \nu_4 = \nu_6 + \nu_4 = \nu_6 + \nu_5 = \nu_3$

$2\nu_9 = \nu_4$

F12-TZ-cCR spectral data of $^{10}BH_2OH$

Table S36: Freqs, ZPT=7767.4 (cm-1)

Mode	HARM	FUND	CORR
1	3869.8	3683.1	3683.1
2	2695.9	2576.3	2576.2
3	2585.7	2474.4	2472.8
4	1416.6	1386.3	1386.0
5	1202.0	1165.5	1165.5
6	1196.6	1175.8	1175.8
7	1079.1	1065.0	1065.0
8	900.5	885.1	885.1
9	789.4	751.0	751.0

Table S37: ABC (MHz)

Constant	Value
A_e	173632.3
B_e	31790.3
C_e	26870.7
A_0	172693.2
B_0	31541.7
C_0	26578.7
A_1	170842.0
B_1	31515.6
C_1	26518.1
A_2	171515.2
B_2	31491.2
C_2	26530.1
A_3	170670.1
B_3	31524.1
C_3	26519.5
A_4	173161.2
B_4	31506.2
C_4	26384.9
A_5	175995.0
B_5	31590.0
C_5	26478.3
A_6	173555.1
B_6	31520.1
C_6	26498.3
A_7	170900.7
B_7	31321.8
C_7	26618.1
A_8	177530.0
B_8	31492.3
C_8	26497.1
A_9	168191.1
B_9	31416.5
C_9	26581.0
K	-0.93207

Table S38: Deltas

Constant	Units	Value
Δ_J	kHz	67.623
Δ_K	MHz	5.431
Δ_{JK}	kHz	540.974
δ_J	kHz	11.582
δ_K	kHz	487.426

Table S39: Phis

Constant	Units	Value
Φ_J	mHz	117.112
Φ_K	Hz	742.389
Φ_{JK}	Hz	11.579
Φ_{KJ}	Hz	-22.451
ϕ_j	mHz	60.031
ϕ_{jk}	Hz	6.742
ϕ_k	Hz	304.809

Table S40: Geom (A or Deg)

COORD	R(EQUIL)	R(ALPHA)
r(H1-O2)	0.9582831	0.9593383
r(O2-B3)	1.3495206	1.3562446
r(B3-H4)	1.1886041	1.1995285
r(B3-H5)	1.1941314	1.2056785
∠(O2-H1-B3)	112.9175285	113.3837626
∠(B3-O2-H4)	116.7901636	116.8328067
∠(B3-O2-H5)	120.4253816	120.5818022

Table S41: Fermi Resonances

$$2\nu_4{=}\nu_5{+}\nu_4{=}\nu_6{+}\nu_4{=}\nu_2$$
$$2\nu_5{=}2\nu_6{=}\nu_5{+}\nu_4{=}\nu_6{+}\nu_4{=}\nu_6{+}\nu_5{=}\nu_3$$
$$2\nu_9{=}\nu_4$$

F12-TZ spectral data of $^{10}BH_3OH_2$

Table S42: Freqs, ZPT=12014.0 (cm-1)

Mode	HARM	FUND	CORR
1	3889.9	3700.9	3700.9
2	3789.2	3612.7	3612.7
3	2601.3	2485.0	2497.9
4	2569.3	2456.8	2456.8
5	2484.1	2379.5	2442.1
6	1653.9	1604.3	1628.6
7	1213.7	1182.8	1183.3
8	1205.3	1177.3	1177.3
9	1200.6	1174.3	1174.7
10	1031.0	968.4	948.8
11	957.2	897.8	902.6
12	647.4	610.3	609.1
13	607.6	543.7	543.7
14	479.1	405.7	406.6
15	156.4	91.9	91.9

Table S43: ABC (MHz)

Constant	Value
A_e	87263.6
B_e	18432.7
C_e	17910.5
A_0	86504.2
B_0	17728.8
C_0	17254.5
A_1	86046.8
B_1	17734.5
C_1	17271.6
A_2	85968.5
B_2	17732.6
C_2	17256.2
A_3	86335.2
B_3	17799.0
C_3	17318.2
A_4	85780.1
B_4	17818.1
C_4	17326.5
A_5	85917.2
B_5	17776.8
C_5	17307.2
A_6	86336.6
B_6	17754.1
C_6	17234.5
A_7	88559.1
B_7	17727.5
C_7	17287.0
A_8	84735.9
B_8	17696.0
C_8	17221.1
A_9	85475.6
B_9	17713.0
C_9	17199.9

Table S44: ABC (MHz, cont.)

Constant	Value
A_{10}	87313.0
B_{10}	17494.8
C_{10}	17013.7
A_{11}	86382.6
B_{11}	17458.2
C_{11}	17030.8
A_{12}	87562.4
B_{12}	17573.5
C_{12}	17066.7
A_{13}	86941.2
B_{13}	17539.9
C_{13}	17064.6
A_{14}	86266.6
B_{14}	17179.9
C_{14}	16733.6
A_{15}	86422.4
B_{15}	17528.9
C_{15}	17171.4
K	-0.98630

Table S45: Deltas

Constant	Units	Value
Δ_J	kHz	98.008
Δ_K	kHz	431.130
Δ_{JK}	kHz	101.684
δ_J	kHz	3.189
δ_K	GHz	-0.001

Table S46: Phis

Constant	Units	Value
Φ_J	Hz	-1.329
Φ_K	Hz	68.756
Φ_{JK}	Hz	16.714
Φ_{KJ}	Hz	-64.668
ϕ_j	mHz	-47.345
ϕ_{jk}	Hz	18.278
ϕ_k	kHz	2.029

Table S47: Geom (A or Deg)

COORD	R(EQUIL)	R(ALPHA)
r(H1-B2)	1.2057530	1.2122810
r(B2-O3)	1.7113675	1.7532254
r(B2-H4)	1.2001106	1.2055238
r(B2-H5)	1.2001106	1.2055238
r(O3-H6)	0.9623613	0.9439403
r(O3-H7)	0.9623613	0.9439403
∠(B2-H1-O3)	102.9894094	102.5272928
∠(B2-H5-O3)	101.3072577	100.9521198
∠(B2-H4-O3)	101.3072577	100.9521198
∠(O3-H6-B2)	108.3323147	109.1280792
∠(O3-H7-B2)	108.3323147	109.1280792

Table S48: Fermi Resonances

$\nu_{14}+\nu_{12}=\nu_{15}+\nu_{11}=\nu_{10}$

$\nu_{15}+\nu_{12}=\nu_{11}$

$\nu_{15}+\nu_{13}=\nu_{12}$

$2\nu_{15}=\nu_{14}$

$\nu_{8}+\nu_{7}=\nu_{9}+\nu_{7}=\nu_{15}+\nu_{4}=\nu_{15}+\nu_{5}=\nu_{3}$

$2\nu_{7}=2\nu_{8}=2\nu_{9}=\nu_{9}+\nu_{8}=\nu_{4}$

$2\nu_{7}=2\nu_{8}=2\nu_{9}=\nu_{9}+\nu_{8}=\nu_{5}$

$\nu_{12}+\nu_{10}=\nu_{13}+\nu_{11}=\nu_{6}$

$\nu_{13}+\nu_{12}=\nu_{14}+\nu_{12}=\nu_{15}+\nu_{11}=\nu_{7}$

$2\nu_{12}=2\nu_{13}=\nu_{8}$

$2\nu_{12}=2\nu_{13}=\nu_{14}+\nu_{13}=\nu_{15}+\nu_{10}=\nu_{9}$

F12-TZ-cCR spectral data of $^{10}BH_3OH_2$

Table S49: Freqs, ZPT=12083.5 (cm-1)

Mode	HARM	FUND	CORR
1	3892.5	3704.9	3704.9
2	3792.0	3615.7	3615.7
3	2608.1	2491.1	2501.6
4	2576.1	2464.3	2464.3
5	2490.6	2386.9	2449.6
6	1655.1	1607.6	1635.3
7	1217.0	1184.1	1184.5
8	1209.0	1180.3	1180.3
9	1204.2	1183.1	1182.7
10	1036.6	980.8	976.5
11	962.4	912.4	930.2
12	651.5	619.3	619.1
13	610.8	528.4	528.4
14	484.4	409.9	408.7
15	158.1	241.1	241.1

Table S50: ABC (MHz)

Constant	Value
A_e	87645.5
B_e	18552.4
C_e	18022.9
A_0	86906.8
B_0	17857.8
C_0	17374.4
A_1	86446.4
B_1	17862.8
C_1	17390.9
A_2	86367.5
B_2	17861.2
C_2	17375.6
A_3	86742.0
B_3	17927.5
C_3	17437.5
A_4	86176.3
B_4	17946.6
C_4	17445.7
A_5	86316.7
B_5	17905.5
C_5	17426.8
A_6	86740.0
B_6	17883.9
C_6	17354.3
A_7	89093.4
B_7	17856.2
C_7	17407.4
A_8	85316.2
B_8	17811.4
C_8	17332.3
A_9	85570.7
B_9	17856.4
C_9	17328.2

Table S51: ABC (MHz, cont.)

Constant	Value
A_{10}	87726.4
B_{10}	17626.7
C_{10}	17136.2
A_{11}	86768.6
B_{11}	17588.8
C_{11}	17152.8
A_{12}	87970.7
B_{12}	17705.3
C_{12}	17188.6
A_{13}	87343.9
B_{13}	17670.6
C_{13}	17186.1
A_{14}	86663.9
B_{14}	17307.8
C_{14}	16852.5
A_{15}	86881.4
B_{15}	17669.8
C_{15}	17301.3
K	-0.98609

Table S52: Deltas

Constant	Units	Value
Δ_J	kHz	97.681
Δ_K	kHz	435.867
Δ_{JK}	kHz	103.765
δ_J	kHz	3.203
δ_K	GHz	-0.001

Table S53: Phis

Constant	Units	Value
Φ_J	Hz	-1.312
Φ_K	Hz	66.278
Φ_{JK}	Hz	15.945
Φ_{KJ}	Hz	-61.191
ϕ_j	mHz	-47.284
ϕ_{jk}	Hz	14.920
ϕ_k	kHz	1.862

Table S54: Geom (A or Deg)

COORD	R(EQUIL)	R(ALPHA)
r(H1-B2)	1.2031473	1.2097045
r(B2-O3)	1.7047509	1.7454899
r(B2-H4)	1.1975300	1.2029580
r(B2-H5)	1.1975300	1.2029580
r(O3-H6)	0.9615372	0.9433821
r(O3-H7)	0.9615372	0.9433821
∠(B2-H1-O3)	103.1117956	102.6955964
∠(B2-H5-O3)	101.4206664	101.0667266
∠(B2-H4-O3)	101.4206664	101.0667266
∠(O3-H6-B2)	108.3895485	109.2659071
∠(O3-H7-B2)	108.3895485	109.2659071

Table S55: Fermi Resonances

$\nu_{14}+\nu_{12}=\nu_{15}+\nu_{11}=\nu_{10}$

$\nu_{15}+\nu_{12}=\nu_{11}$

$\nu_{15}+\nu_{13}=\nu_{12}$

$2\nu_{15}=\nu_{14}$

$\nu_{8}+\nu_{7}=\nu_{9}+\nu_{7}=\nu_{15}+\nu_{4}=\nu_{15}+\nu_{5}=\nu_{3}$

$2\nu_{7}=2\nu_{8}=2\nu_{9}=\nu_{9}+\nu_{8}=\nu_{4}$

$2\nu_{7}=2\nu_{8}=2\nu_{9}=\nu_{9}+\nu_{8}=\nu_{5}$

$\nu_{12}+\nu_{10}=\nu_{13}+\nu_{11}=\nu_{6}$

$\nu_{13}+\nu_{12}=\nu_{14}+\nu_{12}=\nu_{15}+\nu_{11}=\nu_{7}$

$2\nu_{12}=2\nu_{13}=\nu_{8}$

$2\nu_{12}=2\nu_{13}=\nu_{14}+\nu_{13}=\nu_{15}+\nu_{10}=\nu_{9}$

Dipoles

Table S56: Dipole components of HBO. Geometry in Å and dipole in D

B	0.000000000	0.000000000	−0.650107202
O	0.000000000	0.000000000	0.553844168
H	0.000000000	0.000000000	−1.819072092
μ	0.00	0.00	−2.74

Table S57: Dipole components of BH_2OH. Geometry in Å and dipole in D

B	0.000000000	0.018476336	0.745768541
H	0.000000000	−1.011777331	1.349021662
H	0.000000000	1.083942825	1.284394668
O	0.000000000	−0.066896230	−0.604737565
H	0.000000000	0.791546975	−1.032447738
μ	0.00	1.44	−0.45

Table S58: Dipole componenets of BH_3OH_2. Geometry in Å and dipole in D

O	0.000000000	−0.064653700	−0.746454040
B	0.000000000	0.011426424	0.964625279
H	0.000000000	1.197511240	1.159425745
H	−1.020872217	−0.561315547	1.237628875
H	1.020872217	−0.561315547	1.237628875
H	0.771148392	0.414422519	−1.065701023
H	−0.771148392	0.414422519	−1.065701023
μ	0.00	1.42	−3.99
